# Personality and anxiety are related to health-related quality of life in unruptured intracranial aneurysm patients selected for non-intervention: A cross sectional study

**DOI:** 10.1371/journal.pone.0229795

**Published:** 2020-03-12

**Authors:** Mariantonia Lemos, Juan Pablo Román-Calderón, Gabriela Calle, Juan Fernando Gómez-Hoyos, Carlos Mario Jimenez

**Affiliations:** 1 Department of Psychology, School of Humanities, Universidad EAFIT, Medellín, Antioquia, Colombia; 2 Department of International Business, Organization and Management School, Universidad EAFIT, Medellín, Antioquia, Colombia; 3 Hemodynamic Service, Clínica CardioVID, Medellín, Antioquia, Colombia; 4 Neurosurgery Service, Universidad de Antioquia, Medellín, Antioquia, Colombia; Leiden University Medical Center, NETHERLANDS

## Abstract

**Background:**

Personality traits and mental health problems have been previously reported in unruptured intracranial aneurysm (UIA) patients; however, few studies have clarified the relations between these variables and health-related quality of life (HRQoL). This study was designed to characterize the personality traits, HRQoL and mental health of patients with UIA and to evaluate whether personality has an influence on HRQoL and whether this is mediated by the patients' emotional symptoms.

**Methods:**

Sixty-three patients with UIAs (mean age 62.6 years, 83.9% women) answered questionnaires for depression, anxiety, HRQoL and personality traits between June 2016 and May 2019.

**Results:**

Eight percent of the sample had depression, and 27.4% had anxiety. Participants showed high levels of responsibility, kindness and neuroticism and low levels of extraversion and openness. HRQoL scores were normal compared with the Colombian population. Structural equation analysis showed that patients' HRQoL was negatively affected by anxiety levels and that the latter are associated with the patient's personality, where neuroticism is directly associated with symptomatology and inversely associated with extraversion.

**Conclusions:**

The results of this study showed the importance of personality and emotional symptoms in the HRQoL of UIA patients. These results are important for developing strategies for psychological counseling in patients with UIAs.

## Introduction

The presence of unruptured intracranial aneurysms (UIAs) in the general population has been estimated at 3%. Few of these aneurysms rupture, but when they do, the associated mortality is 25–50% [1]. However, taking into account that only between 1% and 3% of these aneurysms actually rupture, the treatment to be followed must consider multiple variables, including the size and location of the aneurysm and the age of the patient. This also includes comorbidities, given that sometimes the best decision is not to perform surgery but, rather, to have the patient attend routine check-ups for management and control [2,3].

The patient’s mere knowledge of having an aneurysm has been found to be associated with lower levels of health-related quality of life (HRQoL). However, there are few studies involving patients who have not undergone surgery after diagnosis [4–6]. A study conducted in the Netherlands involving 21 patients, nine of whom had UIAs, reported low levels of HRQoL compared to the reference population [4]. The same results were found in another study that involved 81 patients diagnosed with UIAs who did not receive intervention [5]. Similarly, a study involving 52 patients that estimated the number of years lost in patients with UIA found that this diagnosis could lead to 0.4 to 1.9 years lost due to the diagnosis. No differences were found between patients with small and large aneurysms [6]. This is important because the improvement of HRQoL is one a public health objective, and health perceptions can be useful to identify unmet needs and help professionals guide interventions with these patients [7].

It is important to note that it is not clear whether HRQoL levels in patients with UIAs improve following intervention [8–10] and that complete functional recovery, return to work and life satisfaction could be affected [11]. A study compared patients who had undergone surgery for an aneurysm but in whom another aneurysm had been left untreated due to its size with patients who had undergone surgery, no longer had any aneurysms; the study showed no significant differences in the HRQoL scales six months after the intervention [8]. Similarly, two studies compared patients who underwent preventive surgery for UIAs with the reference population and found lower HROoL levels in the untreated patients. One of the studies found that all HRQoL subscales were lower, except for pain; these differences were more pronounced in those who had complications during surgery [9]. Another study found differences in role emotional, social functioning and the mental composite score [10]. Along the same lines, a five-year longitudinal study comparing patients with UIAs treated by coiling or clipping and patients being monitored showed that there were no differences in the HRQoL of the patients in the groups [12]. It is worth noting that in this study, the authors reported that 25% of the patients presented symptoms of anxiety at follow-up, whereas 23.8% presented symptoms of depression, emotional symptoms that have been reported previously [13]. Finally, a study with 110 patients who had UIAs and were followed for six years after the intervention found that 19% did not report a complete recovery and 22% did not return to work [11].

It should be noted that the high prevalence of emotional disturbance in people with UIAs may be associated with personality factors. A previous study reported higher levels of neuroticism and very low levels of openness in patients with UIAs compared to patients with meningioma [14,15]. Secondary analyses of this same study indicated that 37.8% of patients with UIAs and 17.2% of those with meningioma had a pre-existing psychiatric history and that, by extracting this population in the analyses, the differences between the samples in terms of HRQoL disappeared [16].

The above data led us to think that personality traits may have an impact on the HRQoL of patients with UIAs and that this relation could be mediated by depression and anxiety levels. Additionally, the impact that the diagnosis of an unruptured intracranial aneurysm has on patients who have decided not to undergo intervention is not clear, and scientific literature is scarce in this regard. Considering that an increasing number of patients are currently diagnosed with this problem, and many of them choose not to undergo the intervention and prefer to live with their aneurysm under the supervision of their treating specialist, it would be very useful to know the levels of anxiety that this diagnosis imposes on them, as well as the impact on their HRQoL. This information could be useful for both the treating neurosurgeon and the multidisciplinary team in charge of this collective of patients, as it could help to develop comprehensive healthcare models that lead these patients to a watchful waiting but at the same time preserve an adequate HRQoL.

We present a descriptive study focused on the analysis of anxiety and its impact on HRQoL, developed as part of a longitudinal study in a cohort of patients who, after a judicious analysis with their treating neurosurgeon, have chosen not to undergo intervention for their aneurysm.

## Materials and methods

### Type of study

Descriptive, correlational, and cross-sectional.

#### Population and sample

The reference population of this study is a sample of patients conforming to an ongoing longitudinal follow-up study in patients with UIAs; they have at least six months of follow-up after the diagnosis was made, usually through catheter-based angiogram or MRI angiography, and they have chosen, after a judicious analysis with their treating neurosurgeon, not to undergo aneurysm intervention but rather to be assigned to watchful waiting. Between June 2016 and March 2019, all the patients who chose conservative management were invited to participate (N = 80); nine of these patients were out of reach, seven refused to participate, and two decided to undergo surgery. Ultimately, 62 patients (77.5% of the original sample) were evaluated.

The mean age was 62.6 years (SD = 12.0, age range 32–82 years), and 83.9% were women. Half of the sample fell within the mid-level socio-economic bracket. The average number of months since diagnosis was 37. 2 (S.D. = 43.4). Other sample characteristics are described in Table 1.

**Table 1:** Characteristics of the sample.

	M (S.D.) / n (%)
Age	62.6 (12.02)
Sex: women	52 (83.9)
Socio-economic position	
Low	9 (15.0)
Medium	31 (51.7)
High	20 (33.3)
Civil status	
Married	25 (40.3)
Separated	9 (14.5)
Widow	15 (24.2)
Single	13 (21.0)
Education	
Primary school	10 (16.1)
Secondary school	21 (33.9)
Technician or technologist	13 (21.0)
Professional	12 (19.4)
Postgraduate	6 (9.7)
Activity	
Employee	23 (37.1)
Unemployed	21 (33.9)
Retired	18 (29.0)
Time since diagnosis (months)	37.24 (43.4)
Presence of chronic illness	53 (86.9)

M: Mean, S.D.: Standard deviation

#### Instruments

The Medical Outcomes Study Questionnaire Short Form-12 (SF-12) was used to assess HRQoL, divided into the physical and mental components [17–19]. We used a Spanish version of the questionnaire, which has shown good levels of validity and reliability [20,21]. The second edition Beck Depression Inventory—BDI-II [22,23] and the Beck Anxiety Inventory–BAI [24,25], both in their Spanish adaptations, were used to assess emotional symptoms. Finally, the Spanish adaptation of the NEO Five-Factor Inventory—NEO-FFI [26,27] was used to evaluate personality traits.

#### Procedure

Patients were invited to participate by their neurosurgeon during their appointments in the months of sample collection (June 2016 –May 2019). Those who agreed to be contacted received the call in the same week in which they had an appointment with their doctor so that the doctor could inform them of the purpose of the study and they could make an appointment for evaluation. Of the patients who were contacted (n = 86), 77.5% (n = 62) signed informed consent forms and completed the evaluation protocol. This study met all legal requirements for human research and was approved by the Ethics Committee at Universidad EAFIT.

#### Statistical analysis

To test the hypotheses of this study, the authors used partial least squares structural equation modeling (PLS-SEM). Smart PLS v 3.2.7 (SmartPLS GmbH, Germany) was selected to conduct the corresponding statistical analyses [28]. The authors posited that anxiety fully mediated the effect of the general factor of personality (GFP) with neuroticism and extraversion as reflective indicators of it. PLS-SEM implies a two-step procedure. First, the measurement model is evaluated. The authors transformed the reversed code of negatively worded times only to test the psychometric properties of the scales. In this step, the internal consistency and validity of the constructs are assessed. To this end, the authors used the criteria recommended by Hair et al. [29].

Constructs’ internal consistency was evaluated using the composite reliability (CR) coefficient. Convergent validity of the constructs was assessed by observing factor loadings and average extracted variance (AVE). In PLS-SEM, the Heterotrait-Monotrait Ratio (HTMT) can be used to evaluate discriminant validity. The second step in the PLS-SEM approach consists of assessing the structural model. In this step, coefficients of determination (R^2^), predictive relevance (Q^2^), size and significance of path coefficients, and effect sizes (f^2^) are observed [30]. Finally, the authors also tested a partial mediation model. This procedure was conducted to verify whether anxiety fully or partially mediated the effect of GFP on HRQoL. Lastly, the researchers controlled for age, sex and time of diagnosis. They also controlled for chronic diseases; to this end, they created a dummy variable (1 = presence of chronic diseases; 0 = absence of chronic diseases). The significance of the specified paths was examined to assess whether the inclusion of these variables affected the model.

## Results

### Descriptive statistics

The characteristics of the aneurysms in the series of patients presented in this paper can be summarized as follows: they are mostly small aneurysms, understood as those smaller than seven mm in diameter; almost all were saccular, and a few had irregularities in the dome, specifically a bleb. In all cases, the decision not to undergo intervention came from the patient, who, in the company of his/her family and after hearing the explanation of his/her treating neurosurgeon, concluded that he/she felt that the risk of the intervention was greater than that of the natural history of the lesion. None of the patients decided to forego intervention because his/her treating neurosurgeon considered the lesion extremely difficult to access or intractable, whether by surgical or endovascular means.

In the test results, 75.6% (95% confidence interval [CI] 61.9 to 89.3) and 63.4% (95% CI 48.0 to 78.8) of the sample presented adequate mental and physical components, respectively. Regarding levels of depression, 16.1% (95% CI 6.71 to 25.6) had mild symptoms, while 8.1% (95% CI 1.1 to 15.0) had moderate and severe symptoms. Mild anxiety was present in 25.8% (95% CI 4.6 to 37.0) of those evaluated, while 12.9% (95% CI 4.3 to 21.5) had moderate symptoms, and 14.5% (95% CI 5.5 to 23.5) had severe symptoms. Subscale measures and HRQoL components indicated positive results for the sample, evidencing the lowest scores in general health. The average scores for depression and anxiety showed a tendency towards anxious but not depressive symptoms. With respect to personality, on average, the patients presented high levels of responsibility, kindness, and neuroticism (80th, 60th and 60th percentile, respectively) in comparison with the Colombian population, as well as low levels of extraversion and openness (35th and 25th percentile, respectively) (Table 2).

**Table 2:** Descriptive statistics of psychological variables.

Variable	M (S.D.)	Range
*Personality traits*		
Neuroticism	52.69 (10.80)	27–75
Extraversion	45.39 (12.41)	25–68
Openness	43.39 (10.08)	25–67
Amability	53.79 (9.61)	27–74
Conscientiousness	49.95 (10.76)	25–75
*Quality of life*		
Physical Functioning	60.48 (35.51)	0–100
Role Physical	59.68 (43.27)	0–100
Role Emotional	71.77 (43.06)	0–100
Vitality	69.03 (29.12)	0–100
Mental Health	71.61 (20.98)	20–100
Social Functioning	71.34 (33.80)	0–100
Pain	72.18 (36.52)	0–100
General Health	50.81 (25.19)	0–100
Physical Component	60.79 (26.18)	6.25–100
Mental Component	67.59 (25.28)	5–100
*Emotional symptoms*		
Beck Anxiety score	13.05 (13.29)	0–65
Beck depression score	9.62 (8.07)	0–33

M: Mean, S.D.: Standard deviation.

### Correlations among personality traits, HRQoL and emotional symptoms

Correlation analysis indicated that there were significant inverse relationships between depression and anxiety with the physical and mental components of HRQoL. At the personality level, relationships were found between neuroticism and the two HRQoL components, as well as with depressive and anxious symptoms. Extraversion was directly related to the mental component and inversely related to depression and anxiety. Finally, an inverse relationship was found between neuroticism and extraversion, and a direct relationship was found between extraversion and openness (Table 3).

**Table 3:** Correlations between subscales.

	NEO-N	NEO-E	NEO-O	NEO-A	NEO-C	PCS	MCS	BAITOT
NEO-E	-0.415**							
NEO-O	0.007	0.376**						
NEO-A	-0.255*	0.223	-0.100					
NEO-C	0.067	-0.093	-0.135	-0.083				
PCS	-0.352**	0.161	0.062	-0.069	0.169			
MCS	-0.402**	0.327*	-0.087	-0.021	0.151	0.536**		
BAITOT	0.509**	-0.420**	0.028	-0.130	-0.082	-0.589**	-0.585**	
BDITOT	0.668**	-0.444**	-0.165	-0.137	-0.073	-0.338**	-0.532**	0.515**

PCS: Physical component summary, MCS: Mental component summary, BAITOT: Beck Anxiety Inventory total score, BDITOT: Beck Depression Inventory total score.

*p < 0.05

** p < 0.01.

### PLS-structural equation modeling

**Fig 1:**
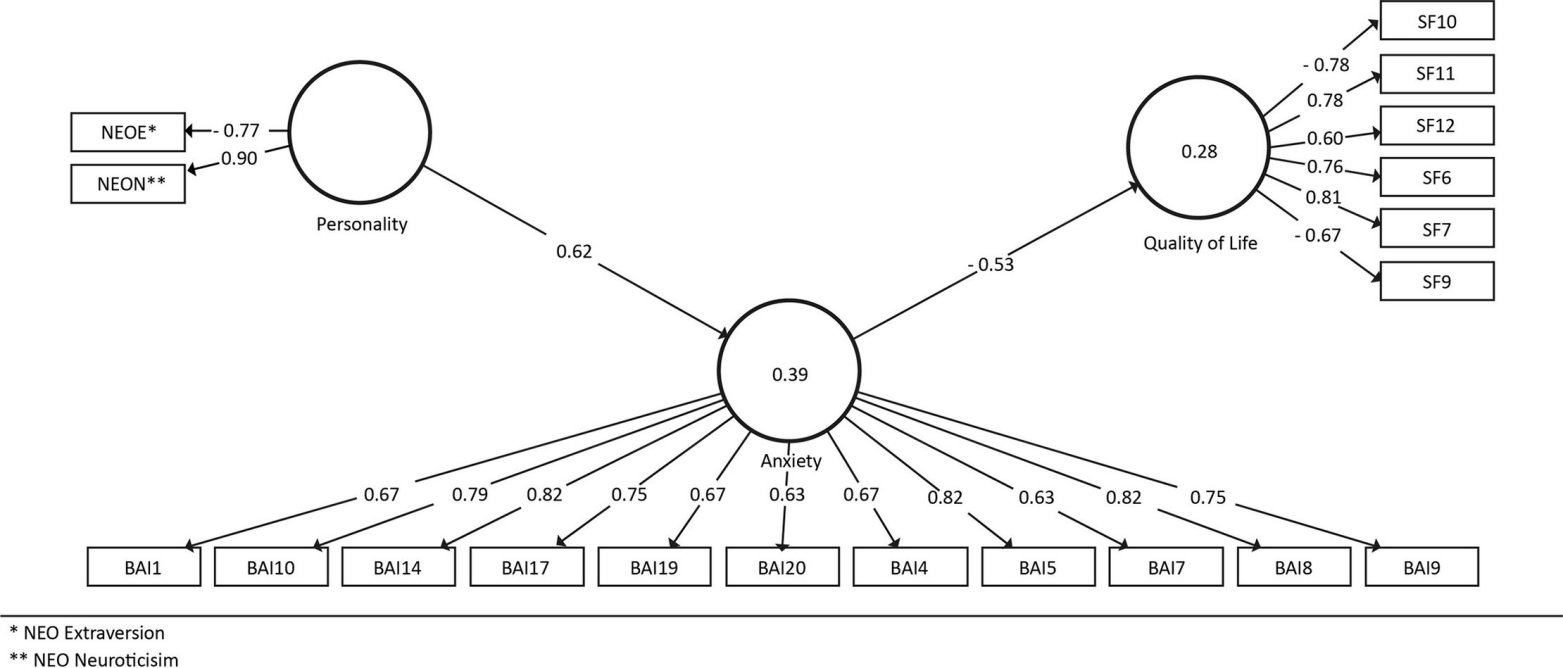
PLS-structural equation model. *NEO Extraversion, ** NEO Neuroticism.

According to the values obtained in the CR, AVE and HTMT coefficients, all the scales were reliable and valid (Table 4). Notably, these scores were obtained after deleting a series of items that did not reach the expected value in terms of factor loadings and for which deletion did not affect the internal consistency of the constructs. Some anxiety and HRQoL items were retained since their deletion affected the internal consistency of the scales, and they almost reached the cut-off value (>0.70) (Fig 1).

**Table 4:** Construct reliability and validity.

			HTMT	
	CR	AVE	1	2
1.Anxiety	0.93	0.54		
2. Quality of Life	0.88	0.54	0.59	
3. GFP	0.82	0.70	0.80	0.67

CR: Construct reliability, AVE: average extracted variance, GFP: General Factor Personality.

Regarding the structural model (see Fig 1), the results support the hypotheses of the study. The coefficients of determinacy were moderate (see Table 5), and the Q2 values were above the cut-off value (Anxiety = 0.18; HRQoL = 0.12). As shown in Fig 1, the effect of GFP on anxiety was positive, with neuroticism loading positively and extraversion negatively on GFP. These two paths were significant (*p* < 0.01 and absence of 0 in confidence intervals). Notably, GFP explained a moderate amount of variance in anxiety, and the corresponding effect size was substantial. Altogether, these findings support H1. Furthermore, H2 was also supported. The results showed that anxiety explained a moderate proportion of HRQoL variance, and the effect size was substantial (see Table 5). With regard to H3, the authors posited that Anxiety mediated the effect of GFP on HRQoL. Accordingly, the indirect effect was negative and significant (β = -0.33, *p* < 0.01, 95% CI -0.46 to -0.20). Furthermore, the direct effect of GFP on HRQoL (i.e. partial mediation model) did not result in significant differences (β = -0.30, *p* = 0.11, 95% CI -0.58 to 0.11). Hence, the results support the hypothesis of total mediation. Finally, after the inclusion of the control variables (i.e. age, sex, time of diagnosis and chronic diseases), all the specified paths remained significant.

**Table 5:** Coefficients of determinacy and effect sizes.

	R^2^		f^2^		
		Classification	1	2	Classification
1. Anxiety	0.39	Moderate		0.39	Substantial
2. Quality of Life	0.28	Moderate			
3. GFP			0.63		Substantial

GFP: General Factor Personality

## Discussion

This study aimed to determine the mental health and HRQoL levels of a group of patients in a Colombian city with UIAs who chose not to undergo intervention for their aneurysm. The study used these data to test a model in which anxiety mediated the effect of GFP on HRQoL. All patients included in the study are part of a longitudinal follow-up cohort study of unruptured aneurysm patients. All patients chose, after a thorough analysis with their treating neurosurgeon and considering the pros and cons of an eventual intervention for their aneurysm, not to undergo the intervention but rather to be assigned to a vigilant follow-up. All of them expressed feeling very aware and confident about the decision they made regarding their aneurysm. For this reason, we consider this group of patients to be free of the acute or sub-acute effect that the diagnosis of the lesion could have on their personality traits and psychological profile.

The results showed that in general, patients had an adequate score for the physical and mental components of HRQoL, with a lower score for the mental component. Regarding mental health, mild symptoms of depression were found in 15.9% of patients, and moderate and severe symptoms were found in 7.9%. Anxiety levels in the sample were higher, with mild levels in 25.4% of those evaluated and clinically significant symptoms in 27.0%. Finally, in terms of personality, the patients evaluated presented high levels of responsibility and low levels of openness.

The bivariate analysis showed that patients' HRQoL is negatively affected by levels of depression and anxiety, as well as neuroticism. Extraversion was negatively related to depressive and anxious symptomatology and directly related to the mental component of HRQoL. Finally, structural equation analysis showed that patients' HRQoL was negatively affected by anxiety levels and that these are associated with the patient's personality, where neuroticism is directly associated with symptomatology and inversely associated with extraversion. Contrary to traditional regressions and analyses of variance that imply the use of composite scores, SEM allows the use of item-level information to test hypotheses regarding latent variables. It also accounts for measurement error present in the items [29], while analyzing composite scores regularly leads to ignoring this source of variance [31]. The use of composite scores has been criticized in applications such as clinical psychology [31]. Thus, this study contributes to research on health psychology by overcoming the use of aggregated scores and their corresponding limitations. The results of the PLS-Structural Equation Model suggest that, through anxiety, GFP indirectly affects the HRQoL of the target population. Moreover, the findings of this study suggest that neuroticism and extraversion exert positive and negative effects on anxiety, respectively. In turn, anxiety negatively impacts HRQoL. A rationale for the different relationship of these two personality dimensions with anxiety is presented hereafter.

These results point to better levels of mental health compared to those reported in China, where the rate of depressive symptoms was 71% and 80% for anxious symptoms [13]. However, these levels are worse than those reported in the two studies conducted in the Netherlands [4,5]. Our data are similar to those found in the study by Li et al. [12] in terms of depressive symptoms but with a higher prevalence of anxious symptoms. This aspect is common in all studies and has also been confirmed in a study that evaluated anxiety before and after surgery in patients with UIAs using the State-Trait Anxiety Inventory. In this study, it was found that state anxiety scores decreased after surgery but that patients had high levels of trait anxiety that remained even after surgery [32]. It should be noted that the differences between the prevalences found may be due to the scales used in each of these studies. Additionally, the sample of patients in the Netherlands was very small, and the majority were not patients with UIAs but with arteriovenous malformations, making it difficult to establish a comparison.

With respect to HRQoL, the results obtained for this sample are similar to those obtained in other studies, evidencing an impact on HRQoL, although not a severe one [4–6]. It is interesting to note that when examining the subscales, the subscale with the lowest score is related to general health, referring to the patients' perception of health. This result was similar to that obtained by the study conducted in the Netherlands and suggests that patients are affected by their diagnosis, even though it does not affect their physical health and is not significantly associated with emotional symptoms or an impairment of their social functioning [4]. These results could be used to hypothesize that the impact of the diagnosis of a UIA affects health perception more than the patients’ physical condition and shows the importance of considering other factors that affect health perception, such as personality and anxiety.

With respect to personality in patients with UIAs, the study conducted in the Netherlands noted high levels of neuroticism in these patients and a history of previous mental disorders. These results suggest that such precursors could be associated with the low HRQoL levels shown and with the differences found with the comparison group [14–16]. However, the study does not establish a relationship between personality traits and the mediation of patient symptomatology, as also found in this study. It should be noted that a systematic review of patients who have already had a hemorrhage associated with an aneurysm indicated that neuroticism is one of the worst prognostic variables for HRQoL [33]. This relationship may also occur in patients who have not suffered a rupture.

Neuroticism is a personality trait that has been described as a relatively stable tendency to respond to situations of frustration, loss, or threat with negative emotions. Individuals with high scores in this trait react to small challenges with intense emotion [34]. Neuroticism has become increasingly important as a precursor to physical and mental health difficulties and, in this case, would be associated with high levels of anxiety that would affect the HRQoL of patients with UIAs. It should be noted that neuroticism by itself is also associated with a decrease in HRQoL even in a normal population, since it includes a sensation of feeling unwell in general, excessive worry, job failure, and little marital satisfaction [35]. On the other hand, people who possess the trait of extraversion tend to be socially inclined, active, assertive, and talkative [26]. This trait is associated with greater positive emotions derived from social contact [36]. In this case, it was found that lower scores in relation to extraversion constitute a personality that reports higher levels of anxiety and, therefore, worse HRQoL.

With respect to the conjunction of neuroticism with extraversion, studies have shown that these can predict affective variability. That is, the extent of an individual's mood variations. This relationship would lead neuroticism to be related mainly to the mean intensity of negative affect and extraversion to the mean intensity of positive affect [37,38]. Similarly, one study noted that subjects with low extraversion but high neuroticism may present greater behavioral inhibition, leading to increased symptoms of anxiety [39]. These results coincide with the model found, suggesting that this personality factor could lead to higher levels of anxiety in patients with UIAs. On this matter, it is important to consider personality factors in the decision process about treatment. Patients with higher levels of neuroticism and low levels of extraversion could have more anxiety about rupture, and those patients could benefit from nonconservative treatment for the aneurysm or be referred to a psychotherapy process [14] oriented to diminishing anxiety levels, which leads to a better HRQoL.

In addition to the model found, it cannot be overlooked that the patients in this study had low levels of openness when compared to the reference population. This becomes important in light of a study involving university students who had to perform a mathematical task as part of an experiment to analyze the cognitive assessment of stress and the influence of their personality [40]. Extrapolating the results to this study's population, it may be considered that a situation such as diagnosis and adjustment to living with a UIA could more likely be viewed as a threat, which increases negative affect, but also that low levels of extraversion reinforce this threat perception. In addition, low openness scores decrease the probability of reducing the perception of threat in such a way that it becomes very likely for the patient to present anxious symptomatology and consequently low HRQoL. The results of this study indicate that in patients with UIAs, variables external to their condition may also affect their HRQoL [41]. These findings also support the initiative of other authors who have proposed that it is necessary to encourage an optimistic attitude and social activity in these patients [13], especially for those in whom anxious symptoms and neurotic traits are found [15].

Some methodological aspects need to be discussed. Although there has been controversy around the existence of GFP, a recent meta-analytic study supported its existence [42]. Additionally, the use of PLS-SEM was determined because this analysis is suitable to test theoretical models implying relationships between latent variables without making assumptions such as normality of distributions and with relatively small sample sizes [29]. Regarding this point, our sample size is similar to previous studies; however, we found that there are only a few studies of patients with UIA who did not receive intervention. This type of study is important to understand the mental and physical HRQoL of these patients, a variable that must be considered to decide the best treatment for each patient with this condition. Additionally, in this study, we collected 63 observations. This sample size is above the sample size recommended by Hair and colleagues [29] to obtain R square values of 0.50 with a 1% significance level and a statistical power of 80%.

These results showed that HRQoL patients are affected by the diagnosis of UIAs. These results have been found previously [4–6]. It is important to note that this affectation has been spotted in patients who have undergone intervention [8–11], which leads us to hypothesize that there are other factors that contribute to lower health and wellbeing perceptions in these patients. This study showed that HRQoL is affected by anxiety levels and is related to personality factors. We cannot affirm that this is only true for individuals with UIAs, but this gives us some ideas of the specific targets needed to intervene in a psychotherapeutic process with this population. Even when personality has been shown to be a stable construct, psychotherapies such as cognitive behavioral therapy [43,44] could focus on giving patients the skills to perceive this diagnosis as less threatening, to assess and evaluate anxiety-specific thoughts and to engage in healthier habits that maintain good levels of HRQoL.

Finally, we must consider the limitations of this study. The first is that the sample of patients was small, which means that the final model could not include more variables that would have been interesting to analyze, such as the influence of time since the diagnosis. However, the correlations established over time did not appear to be significant in the bivariate analyses. A second point to consider is that self-report tests were used for the diagnosis of depression and anxiety, and this information was not confirmed by clinical interviews. This can lead to higher prevalences due to the sensitivity of the tests. Third, the SF-12 is a general questionnaire of HRQoL that examines a limited number of domains that contribute to QoL in patients. The use of a disease-specific QoL questionnaire is recommended for future studies. Fourth, and perhaps most importantly, neuroticism measures are not independent of those for depression and anxiety, meaning that the relationship between personality and anxiety should be considered with caution, although the causal relationship of neuroticism to emotional symptoms has even been proven in longitudinal studies.

## Conclusions

This study indicates that patients with UIAs, even those with a relatively long period after diagnosis, who have judiciously decided not to undergo intervention but rather to undergo vigilant follow-up, have significantly higher levels of anxiety compared to the reference population, as well as some deterioration in their HRQoL, especially in their perception of health. Regarding personality, it was found that these patients have low levels of openness and moderate levels of neuroticism. Finally, it was found that HRQoL levels are mediated by anxiety symptoms and that anxiety is associated with personality, understood as high neuroticism and low extraversion. These results may be the basis for developing strategies for psychological counseling in patients with UIAs as part of a multidisciplinary approach that actively involves the treating neurosurgeon, if it is intended to positively impact the quality of life of this group of patients, which could eventually be part of a preventive model aimed at mitigating the risk of aneurysm rupture.

## Supporting information

S1 Dataset (XLSX)

## References

[1] Kang H, Peng T, Qian Z, Li Y, Jiang C, Ji W, et al Impact of hypertension and smoking on the rupture of intracranial aneurysms and their joint effect. Neurol Neurochir Pol. 2015;49: 121–125. 10.1016/j.pjnns.2015.03.005 25890927

[2] Damani R, Mayer S, Dhar R, Martin RH, Nyquist P, Olson DM, et al Common data element for unruptured intracranial aneurysm and subarachnoid hemorrhage: recommendations from assessments and clinical examination workgroup/subcommittee. Neurocrit Care. 2019;30: 28–35. 10.1007/s12028-019-00736-1 31090013

[3] Hackenberg KAM, Algra A, Salman R, Frösen J, Hasan D, Juvela S, et al Definition and prioritization of data elements for cohort studies and clinical trials on patients with unruptured intracranial aneurysms: proposal of a multidisciplinary research group. Neurocrit Care. 2019;30: 87–101. 10.1007/s12028-019-00729-0 31102238

[4] Van der Schaaf IC, Brilstra EH, Rinkel GJE, Bossuyt PM, Van Gijn J. Quality of life, anxiety, and depression in patients with an untreated intracranial aneurysm or arteriovenous malformation. Stroke. 2002;33: 440–443. 10.1161/hs0202.102335 11823649

[5] Buijs JE, Greebe P, Rinkel GJE. Quality of life, anxiety, and depression in patients with an unruptured intracranial aneurysm with or without aneurysm occlusion. Neurosurgery. 2012;70: 868–872. 10.1227/NEU.0b013e3182367295 21937934

[6] Yoshimoto Y, Tanaka Y. Risk perception of unruptured intracranial aneurysms. Acta Neurochir (Wien). 2013;155: 2029–2036. 23921577 10.1007/s00701-013-1829-3

[7] Centers-for-Disease-Control-and-Prevention. Health-related quality of life (HRQOL). National Center for Chronic Disease Prevention and Health Promotion, Division of Population Health. 2018 [cited 2019 January 11]. Available from: https://www.cdc.gov/hrqol/concept.htm

[8] Van der Schaaf IC, Wermer MJH, Velthuis BK, Buskens E, Bossuyt PMM, Rinkel GJE. Psychosocial impact of finding small aneurysms that are left untreated in patients previously operated on for ruptured aneurysms. J Neurol Neurosurg Psychiatry. 2006;77: 748–752. 10.1136/jnnp.2005.079194 16705198 PMC2077475

[9] Pala A, Pawlikowski A, Brand C, Schmitz B, Wirtz CR, König R, et al Quality of life after treatment of unruptured intracranial aneurysms. World Neurosurg. 2019;121: e54–e59. 10.1016/j.wneu.2018.09.010 30244183

[10] Dammann P, Wittek P, Darkwah-Oppong M, Hütter BO, Jabbarli R, Wrede K, et al Relative health-related quality of life after treatment of unruptured intracranial aneurysms: long-term outcomes and influencing factors. Ther Adv Neurol Disord. 2019;12: 1756286419833492 10.1177/1756286419833492 30886649 PMC6410394

[11] Backes D, Rinkel GJE, van der Schaaf IC, Bijvank NJA, Verweij BH, Visser-Meily JMA, et al Recovery to preinterventional functioning, return-to-work, and life satisfaction after treatment of unruptured aneurysms. Stroke. 2015;46: 1607–1612. 10.1161/STROKEAHA.115.008795 25922514

[12] Li Y, Dai W, Zhang J. Anxiety, depression and quality of life in patients with a treated or untreated unruptured intracranial aneurysm. J Clin Neurosci. 2017;45: 223–226. 10.1016/j.jocn.2017.07.019 28778800

[13] Su S-H, Xu W, Hai J, Yu F, Wu Y-F, Liu Y-G, et al Cognitive function, depression, anxiety and quality of life in Chinese patients with untreated unruptured intracranial aneurysms. J Clin Neurosci. 2014;21: 1734–1739. 10.1016/j.jocn.2013.12.032 24913931

[14] Wenz H, Wenz R, Ehrlich G, Groden C, Schmieder K, Fontana J. Patient characteristics support unfavorable psychiatric outcome after treatment of unruptured intracranial aneurysms. Acta Neurochir (Wien). 2015;157: 1135–1145; discussion 1145. 26007696 10.1007/s00701-015-2451-3

[15] Wenz H, Wenz R, Maros ME, Groden C, Schmieder K, Fontana J. The neglected need for psychological intervention in patients suffering from incidentally discovered intracranial aneurysms. Clin Neurol Neurosurg. 2016;143: 65–70. 10.1016/j.clineuro.2016.02.018 26896784

[16] Fontana J, Wenz R, Groden C, Schmieder K, Wenz H. The preinterventional psychiatric history as a major predictor for a reduced quality of life after treatment of unruptured intracranial aneurysms. World Neurosurg. 2015;84: 1215–1222. 10.1016/j.wneu.2015.06.047 26142812

[17] Gandek B, Ware JE, Aaronson NK, Apolone G, Bjorner JB, Brazier JE, et al Cross-validation of item selection and scoring for the SF-12 health survey in nine countries: results from the IQOLA project. International quality of life assessment. J Clin Epidemiol. 1998;51: 1171–1178. 10.1016/s0895-4356(98)00109-7 9817135

[18] Jenkinson C, Layte R, Jenkinson D, Lawrence K, Petersen S, Paice C, et al A shorter form health survey: can the SF-12 replicate results from the SF-36 in longitudinal studies? J Public Health Med. 1997;19: 179–186. 10.1093/oxfordjournals.pubmed.a024606 9243433

[19] Ware JE, Keller SD, Kosinski M. SF-12: how to score the SF-12 physical and mental health summary scales 2nd ed. Boston, MA: Health Institute, New England Medical Center; 1998.

[20] Vilagut G, Valderas JM, Ferrer M, Garin O, López-García E, Alonso J. Interpretación de los cuestionarios de salud SF-36 y SF-12 en España: componentes físico y mental. Med Clin. 2008;130: 726–735. 10.1157/13121076 18570798

[21] Ramírez-Vélez R, Agredo-Zuñiga RA, Jerez-Valderrama AM. Confiabilidad y valores normativos preliminares del cuestionario de salud SF-12 (short form 12 health survey) en adultos Colombianos. Rev Salud Publica. 2010;12: 807–819. 21755108

[22] Beck AT, Steer RA, Brown GK. Inventario de depresión de Beck II—manual. Madrid: Pearson Clinical & Talent Assessment España; 2011.

[23] Beck AT, Ward CH, Mendelson M, Mock J, Erbaugh JK. An inventory for measuring depression. Arch Gen Psychiatry. 1961;4: 561–571. 10.1001/archpsyc.1961.01710120031004 13688369

[24] Beck AT, Steer RA. Inventario de ansiedad de Beck—manual Madrid: Pearson Clinical & Talent Assessment España; 2011.

[25] Sanz J, Navarro ME. Propiedades psicométricas de una versión española del inventario de ansiedad de Beck (BAI) en estudiantes universitarios. Ansiedad y Estres. 2003;9: 59–84.

[26] Costa PT Jr, McCrae RR. Inventario de personalidad NEO revisado (NEO PI-R) Inventario NEO reducido de cinco factores (NEO-FFI). Manual profesional. 3rd ed. Madrid: TEA Ediciones; 2008.

[27] Manga D, Ramos F, Morán C. The Spanish norms of the NEO five-factor inventory: new data and analyses for its improvement. Int J Psychol Psychol Ther. 2004;4: 639–648.

[28] Ringle CM, Wende S, Bender JM. SmartPLS 3. Boenningstedt: SmartPLS GmbH; 2015.

[29] Hair JF Jr, Hult GTM, Ringle C, Sarstedt M. A primer on partial least squares structural equation modeling (PLS-SEM) 2nd ed. Thousand Oaks: Sage Publications; 2016.

[30] Cohen J. Statistical power analysis for the behavioral sciences Mahwah, NJ: Lawrence Erlbaum; 1988.

[31] Marsh HW, Morin AJ, Parker PD, Kaur G. Exploratory structural equation modeling: an integration of the best features of exploratory and confirmatory factor analysis. Annu Rev Clin Psychol. 2014;10: 85–110. 10.1146/annurev-clinpsy-032813-153700 24313568

[32] Otawara Y, Ogasawara K, Kubo Y, Tomitsuka N, Watanabe M, Ogawa A, et al Anxiety before and after surgical repair in patients with asymptomatic unruptured intracranial aneurysm. Surg Neurol. 2004;62: 28–31; discussion 31. 10.1016/j.surneu.2003.07.012 15226063

[33] Passier PECA Visser-Meily JMA, Rinkel GJE, Lindeman E, Post MWM. Determinants of health-related quality of life after aneurysmal subarachnoid hemorrhage: a systematic review. Qual Life Res. 2013;22: 1027–1043. 22956388 10.1007/s11136-012-0236-1

[34] Lahey BB. Public health significance of neuroticism. Am Psychol. 2009;64: 241–256. 10.1037/a0015309 19449983 PMC2792076

[35] Widiger TA, Oltmanns JR. Neuroticism is a fundamental domain of personality with enormous public health implications. World Psychiatry. 2017;16: 144–145. 10.1002/wps.20411 28498583 PMC5428182

[36] Pang Y, Cui Q, Wang Y, Chen Y, Wang X, Han S, et al Extraversion and neuroticism related to the resting-state effective connectivity of amygdala. Sci Rep. 2016;6: 35484 10.1038/srep35484 27765947 PMC5073227

[37] Dauvier B, Pavani JB, Le Vigouroux S, Kop JL, Congard A. The interactive effect of neuroticism and extraversion on the daily variability of affective states. J Res Pers. 2019;78: 1–15.

[38] Verduyn P, Brans K. The relationship between extraversion, neuroticism and aspects of trait affect. Pers Individ Dif. 2012;52: 664–669.

[39] Klinger-König J, Hertel J, Terock J, Völzke H, Van der Auwera S, Grabe HJ. Predicting physical and mental health symptoms: additive and interactive effects of difficulty identifying feelings, neuroticism and extraversion. J Psychosom Res. 2018;115: 14–23. 10.1016/j.jpsychores.2018.10.003 30470312

[40] Schneider TR, Rench TA, Lyons JB, Riffle RR. The influence of neuroticism, extraversion and openness on stress responses. Stress Health. 2012;28: 102–110. 10.1002/smi.1409 22281953

[41] Solheim O, Eloqayli H, Muller TB, Unsgaard G. Quality of life after treatment for incidental, unruptured intracranial aneurysms. Acta Neurochir (Wien). 2006;148: 821–830; discussion 830. 16791435 10.1007/s00701-006-0804-7

[42] Davies SE, Connelly BS, Ones DS, Birkland AS. The general factor of personality: the “Big One,” a self-evaluative trait, or a methodological gnat that won’t go away? Pers Individ Dif. 2015;81: 13–22.

[43] Beck JS. Cognitive behavior therapy Basics and beyond. 2nd ed. New York: The Guilford Press; 2011.

[44] Clark DA, Beck AT. Cognitive therapy of anxiety disorders: science and practice New York: Guilford Press; 2010.

